# Strengthening Social Capital to Address Isolation and Loneliness in Long-Term Care Facilities During the COVID-19 Pandemic: Systematic Review of Research on Information and Communication Technologies

**DOI:** 10.2196/46753

**Published:** 2023-08-14

**Authors:** Idrissa Beogo, Drissa Sia, Stephanie Collin, Andi Phaelle Gedeon, Michaël-Christopher Louismé, Jean Ramdé, Marie-Pierre Gagnon, Eric Tchouaket Nguemeleu

**Affiliations:** 1 University of Ottawa Ottawa, ON Canada; 2 Département des sciences infirmières Université du Québec en Outaouais Saint-Jérôme, QC Canada; 3 Université de Moncton Moncton, NB Canada; 4 Université Laval Quebec, QC Canada

**Keywords:** information and communication technology, ICT, long-term care facilities, COVID-19, social isolation, loneliness, pandemic, implementation sciences, protocol, nursing home, mobile phone

## Abstract

**Background:**

The COVID-19 pandemic has disproportionately and severely affected older adults, namely those living in long-term care facilities (LTCFs). Aside from experiencing high mortality rates, survivors were critically concerned by social isolation and loneliness (SIL). To address this serious public health concern and stay connected with LTCF residents, information and communication technology (ICT) platforms (eg, video calls) were used as an alternative to maintaining social interactions amid the visiting restriction policy.

**Objective:**

This paper aimed to synthesize the effects of ICT-related communication interventions using SMS text messaging or chat, video, voice mail, or photo to address SIL in LTCF residents during the COVID-19 pandemic.

**Methods:**

In total, 2793 references published in English and French in 2019 and onward were obtained from 10 relevant databases: PsycINFO-Ovid, Ovid-MEDLINE, CINAHL-EBSCO, Cochrane Library, Web of Science, Scopus, DirectScience, Communication & Mass Media Complete, IEEE Xplore, and ACM Digital Library. A 2-person screening approach was used, and the studies were screened independently and blindly. A narrative synthesis was performed to interpret the results of the included studies, and their quality was appraised.

**Results:**

In total, 4 studies were included in the review. ICT-related applications were used to ensure connectedness to address SIL. ICT interventions consisted mainly of videoconferencing, intergroup video call sessions between residents, and chatting (SMS text messages and phone calls). Roughly 3 classes of mediating ICT tools were used: video calls using software applications (eg, Skype); robot systems embedding video telephones; and ordinary telecommunication such as telephone, internet, social media platforms, and videoconferencing. This review has included the role of humanoid robots in LTCFs as an innovation avenue because of their multipurpose use (eg, communication tools and remotely operable).

**Conclusions:**

Remote social capitalization through ICT applications has become an avenue to reduce SIL among LTCF residents. This review examined a social connection approach that will remain relevant and even be fostered after the COVID-19 pandemic. As families remain the main stakeholders of LTCFs, this study’s findings could inform policy makers and frontline managers to better shape programs and initiatives to prevent or reduce SIL in LTCFs.

**International Registered Report Identifier (IRRID):**

RR2-10.2196/36269

## Introduction

### Background

The COVID-19 pandemic has caused extreme loss of life, especially in long-term care facilities (LTCFs). We can acknowledge that this global crisis disrupted the entire health care system sector [1]. In Canada, LCTFs house 6.8% of people aged ≥65 years [2]. Health care systems experienced an unprecedented crisis. Approximately 80% of deaths occur in the long-term care (LTC) sector, with Canada being among the worst performers, with a rate double the average death rate of the Organization for Economic Co-operation and Development countries’ mortality (42%) [3]. In response to the spread of the COVID-19 pandemic, tight visitation restrictions were levied, isolating the LTCFs from the rest of the community, subsequently isolating older adults, already fraught with the morbidity of social isolation and loneliness (SIL) [4,5]. These measures removed the main sources of support (care, social, etc) provided in the pre–COVID-19 era by informal family caregivers (FCs). Consequently, to address the SIL induced by the lockdown on older adults, almost all previous in-person interactions with older adults were replaced by web-based chatting using information and communication technology (ICT) applications, such as FaceTime, Zoom, and Microsoft Teams video chatting.

A wealth of literature has demonstrated that SIL is negatively associated with several adverse outcomes, such as teeth loss acceleration as reported in a longitudinal study [6], risk of premature mortality [7], and mental health disorders (eg, depression and anxiety) [8], to cite a few. Before the pandemic, the importance of families’ partnership with the health care sector was well-documented. Strong family or community support is essential to maintain social ties with families, friends, and the community at large [9,10]. Approximately one-fifth of families directly care for their older adults [11]. The pandemic has been associated with worsening SIL, which preexisted at a rate of ≥40% [4,5]. It also appears that most older adults are unenthusiastic about leaving their community home to move to an LTCF [12,13]. Many residents who experience marginalization associated with sexual orientation—4% of the general population [14]—or race (Black, Asian, etc) or religion (eg, Muslim) possibly faced more SIL during the pandemic.

ICT applications have been overwhelmingly used as an innovative alternative avenue to stay in touch with loved ones and bolster resilience to increasing SIL worsened by the pandemic’s restrictions. Therefore, families substituted their traditional face-to-face visits with virtual visits (video calls, phone calls, chat, or photo posting) [15,16]. This initiative was welcomed, although the Canadian system’s LTCFs are poorly equipped with IT infrastructure. Indeed, LTCFs operate outside of the Canada Health Act, which provides universal access, public funding, and transferable physician care in hospitals [17]. In contrast, LTCFs operate to meet social needs as opposed to medically necessary needs. Social activities (bingo, painting, outings, walks, etc) that were traditionally conducted in person [18] were consequently suspended during most of the pandemic. In addition, some authors have stated that video interactions showed effectiveness in terms of learning, and stimulation of cognitive activity [19] as well as knowledge transfer [20]. ICT has emerged as a promising, new, and innovative avenue for maintaining social connections. Unfortunately, some studies highlight the lack of technological literacy among older people and their families [21]. The use of ICT to reduce SIL has been extensively studied, even in the pre–COVID-19 pandemic era [22,23]. These studies included the effects of internet-based interventions [24-26] and humanoid robot approaches [27]. However, systematic reviews on remote interventions are still undecided regarding the positive outcomes of social loneliness. Nevertheless, we have witnessed an increased use of ICT applications—from the conventional telephone to web-based platforms, such as Skype, FaceTime, Zoom, or Google Meet. Banskota et al [28] have identified 15 types of applications—social networking (FaceTime and Skype), medical telemedicine (Teladoc, K health, and Doctor on Demand), medical prescription management (GoodRX and Medisafe Medication Management), health and fitness (Calm, Headspace Medication and Sleep, Yoga Down Dog, and MyFitnessPal), food and drink (DoorDash and Instacart), and visual and hearing impairment (Be My Eyes Helping the Blind and Glide)—that older adults have used during the COVID-19 pandemic. Chen and Schulz [29] underscored the ones used by families to interact with their loved ones in LTCFs as well as with the LTCF staff members because of the rising incidence of COVID-19 cases and associated visitation restrictions.

As the population ages worldwide and the family size decreases, SIL is becoming an increasingly pertinent public health issue. In Canada, according to the International Federation on Aging, keeping older people socially connected and active is a challenge [30]. In the past decade, many high-income countries have implemented large-scale programs to address the growing concern that SIL poses in societies [31-35]. The estimated cost of voluntary work by FCs a decade ago was valued at approximately CAD $25-$26 billion in Canada (a currency exchange rate of CAD $1=US $0.76 is applicable) [36]. In a 2012 study, 22% of caregivers provided 10 hours a week of personal care for older adults in collective dwellings [11]. This shows how FCs remain an essential labor force in the health care system. For example, for older Canadian adults living with complex conditions, frailty, and impairments, FCs provide up to 70% to 90% of the care [37].

Before the COVID-19 pandemic, reviews were conducted to demonstrate the impact of ICT on addressing SIL in LTCFs. Although the most recent Cochrane rapid review [22] did not prove a conclusive advantage, an earlier systematic review [29,38] on the use of ICT to combat SIL concluded that ICT was effective at reducing SIL in older adults. In the context of COVID-19, the use of commercial ICT has peaked in the general population as well as in LTCFs to sustain social connections and address SIL [39]. This was observed in various ways. On the one hand, older adults want to learn and strengthen their ability to use social media platforms to keep themselves connected to the outside world, meanwhile, some LTCFs have created ICT-mediated internet-based platforms (eg, Facebook to keep connected with their residents) to maintain vital interactions. Finally, some governments have provided programs and utility hardware (iPad). In Quebec, for instance, public LTCFs have implemented a budget to allow health network managers to purchase iPad devices for telemedicine, remote social interaction, and other psychoeducational activities.

### Research Questions and Objectives

This review intends to assess the effects of ICT interventions implemented in LTCFs to address SIL among residents during the COVID-19 pandemic. The following objectives will be considered to address the research question:

1. To synthesize the effects of ICT-related communication interventions to address SIL in LTCF residents during the COVID-19 period.
2. To identify studies that use ICT, namely through various means of communication, such as texting or chat, video, voice mail, or photo, as a strategy for interaction and connection with older family members living in LTCFs.

## Methods

### Overview

We used a comprehensive and current database to catalog the literature on the use of ICT interventions to address SIL during the COVID-19 pandemic crisis. This systematic review considered world literature, and the selected criteria were based on a scrutiny framework (Population, Interventions, Comparators and designs, Outcomes [PICO]) and a robust search strategy. The review protocol was registered at Open Science Framework [40]. The PRISMA (Preferred Reporting Items for Systematic Reviews and Meta-Analyses) checklist was implemented to provide all relevant information related to the systematic review [41]. We adhered to the Cochrane Collaboration guidelines [42]. The Synthesis Without Meta-analysis guideline [43] was consulted to guide the use of alternative synthesis methods.

### Types of Participants

This systematic review included studies dealing with older adults living in any form of congregate institutional arrangement settings, such as nursing homes, municipal homes for older adults, and charitable homes for older adults. As per the inclusion criteria, the study should include people aged ≥65 years without severe mental illness.

### Inclusion and Exclusion Criteria

Inclusion and exclusion criteria were based on the PICO framework.

#### Population

The inclusion criterion was studies including participants aged ≥65 years and those residing in an LTCF that is a congregate institutional arrangement setting (eg, nursing homes or assisted living arrangements). Exclusion criteria included studies of people (1) with a terminal illness, (2) who were hospitalized, (3) who had severe neurocognitive disorders, (4) with severely impaired cognition (measured by specific tools, such as the Mini-Mental State Examination) [44], or (5) who were community dwellers.

#### Intervention

As this systematic review targets the use of information technology applications, we included video, voice mail, photo, or any form of chat using any commercial applications (eg, TikTok, FaceTime, Facebook, and Zoom) for conversation through a digital tool (eg, computers, smartphones, or tablets). These accommodations help maintain or improve the connection between families and their older loved ones residing in an LTCF with the ultimate goal of combating SIL. The main ICT intervention component had to involve the use of the internet to fulfill social networking needs. We also considered the standard telephone use. The interventions had to be delivered individually or in a collective format. We excluded any form of digital accommodation requiring an important face-to-face discussion component or telecommunication for medical assessment and treatment.

#### Comparator and Designs

This study included quantitative design studies, namely quasi-experimental studies, cohort studies, cross-sectional studies, randomized controlled trials, quasi–randomized controlled trials, and pre-post intervention studies. Qualitative and mixed methods studies were also targeted. Studies that compared ICT interventions with alternative interventions, such as visits through windows or contactless control groups during the pandemic, were included. As planned in the protocol [45], all ICT-based therapeutic interventions such as telehealth or telemedicine, as defined by the World Health Organization [46], were also excluded. Further comparisons among virtual technology–enabled groups such as telephone versus video calls were included.

#### Outcome

##### Primary Outcomes

The primary outcomes were as follows:

1. Measures of SIL (ie, scores on any qualitative appropriation)
2. Measures of SIL through proxy outcomes (ie, the lack of companionship, the lack of friendship, a feeling of being forgotten and not belonging, and the lack of connection with family)

##### Secondary Outcomes

The secondary outcomes were as follows:

1. Self-reported measures of symptoms of depression (ie, scores on any self-report questionnaire designed to quantify the severity of depression symptoms)
2. Self-reported measures of quality of life (QOL; ie, scores on any self-report questionnaire designed to allow people to rate the QOL either overall or within specific domains)

#### Timeline

This study covered publications completed since the onset of the COVID-19 pandemic from December 2019 onward.

### Data Source and Search Strategy

An exploratory search of the MEDLINE database was first performed to scan for titles, abstracts, keywords, and descriptors. Subsequently, the complete search strategy was developed to shape each database’s specificities accordingly. We used an iterative process to enhance the search strategy and adjusted the search results for each database to help capture all potential studies.

Multimedia Appendix 1 presents the final search strategy that consists of the following databases: PsycINFO-Ovid, Ovid-MEDLINE, ACM Digital Library, CINAHL-EBSCO, Cochrane Library, Web of Science, Scopus, DirectScience, Communication & Mass Media Complete, and IEEE Xplore.

The final search of the Chinese databases planned in the protocol was inconclusive (CNKI, WanFang, Weipu [VIP], and SinoMed). Finally, we only considered studies published in French and English from these databases.

### Study Selection and Data Extraction

**Figure 1:**
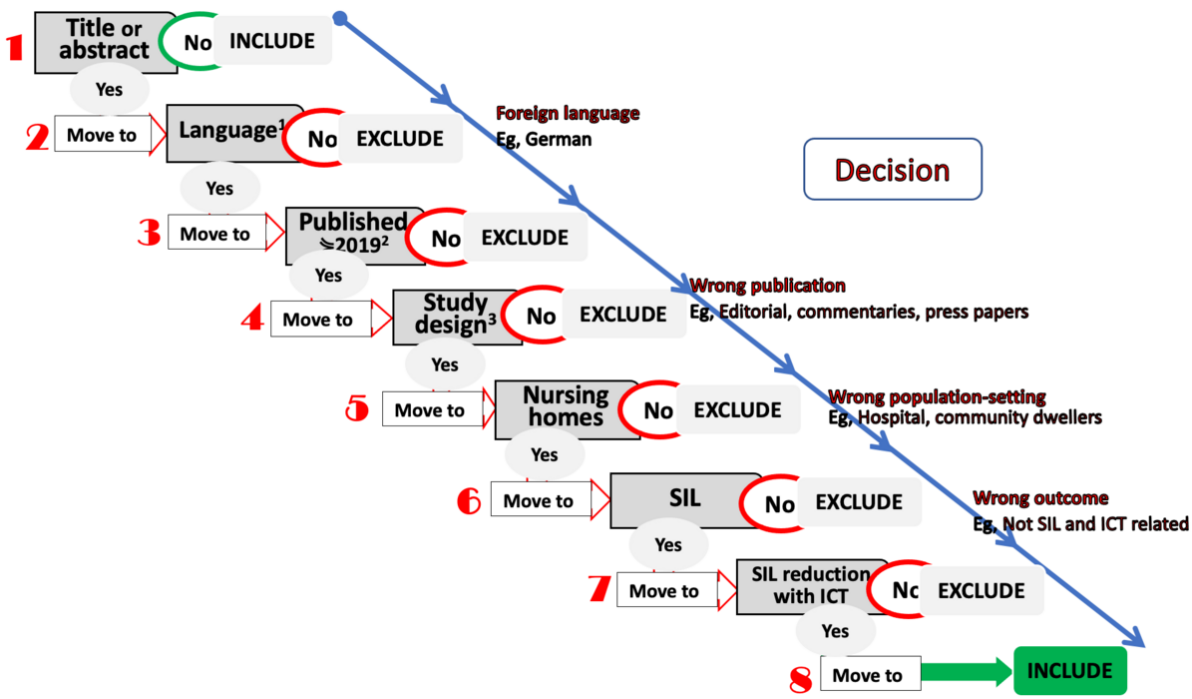
Screening Algorithm.

All records from the queried databases were first transferred to the EndNote package, where duplicates were removed electronically and manually and then transferred to the Rayyan web platform [47]. We implemented a 2-person screening approach, in which 2 research assistants and coauthors (APG and MCL) independently screened the titles and abstracts of potentially eligible studies. A structured algorithm (Figure 1) developed and implemented by IB, DS, and ETN in previous studies was used to support the process. Finally, conflicts were solved by the principal investigator (PI) IB.

### Data Extraction

APG proceeded with data extraction under the supervision of the PI. This was preceded by a pilot test with the PI. A Microsoft Excel spreadsheet was used to analyze the data extracted. For each publication included, systematic data extraction was performed to summarize (1) the objectives, (2) the intervention objectives, (3) the participants’ characteristics, (4) the experimentations (technology used and duration), (5) the method of the study (study design, inclusion of a control group, and the assessment tools used), (6) the barriers and enablers to the implementation of the technology, (7) the benefits of the intervention on social interactions, and (8) the solutions to overcome barriers to the implementation of the technology (Multimedia Appendix 2).

### Data Analysis and Synthesis

The analysis consisted of identifying relevant data or segments of data linked to the objective of the review in each article. For qualitative-like study used, findings were organized according to the purpose of the use of ICT-related applications, the feelings of older adults regarding the applications, and their impact on their relatives. A thematic approach was used to identify themes from the presented data. Because the study explored a better understanding of users’ experiences, we used a descriptive phenomenological approach [48] and thematically dealt with data analysis because of the high flexibility of this approach [49]. We considered inductive coding to determine the final themes [50]. A recurring emergent pool of subthemes derived from data was consensually retained as main themes, either naturally or aggregated based on the objectives and prevalent literature [51]. No theme categories were preset. Finally, 6 main themes were considered: (1) the focus area of the technology, (2) the use of the temi robot (Medisana GmbH), (3) the use of ordinary telephone versus the use of video call, (4) virtual remote communication, (5) effects of technology on SIL, and (6) effects of technology on other outcomes. Analyses were performed manually. Subsequently, a thematic analysis was performed.

### Ethics Approval

As this systematic review is part of the Social Isolation and Loneliness project, we received ethics approval from the Ethics Committees for Research of the University of Ottawa (H-08-21-7314), the University of Moncton (dossier 2021-073), and the Research Ethics Board of the Primary Care and Population Health Research Sector of the Centre intégré universitaire de santé et de services sociaux of the Capitale-Nationale (2021-2303_SPPL).

## Results

### Overview

**Figure 2:**
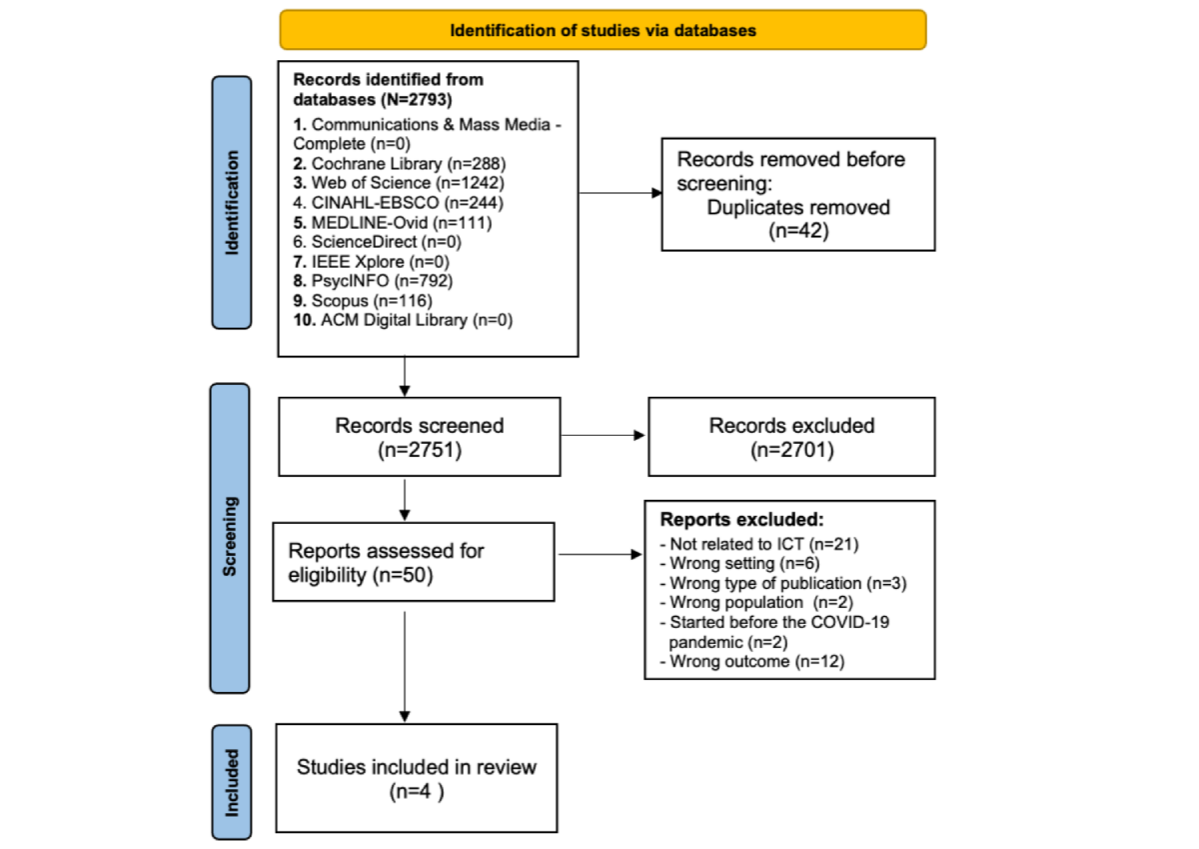
PRISMA (Preferred Reporting Items for Systematic Reviews and Meta-Analyses) flow diagram illustrating the search strategy. ICT: information and communication technology.

The flow diagram illustrated in Figure 2 illustrates the selection flow of the initial yield of 2793 articles. The screening of titles in the abstracts led to 49 studies. Their plain texts were read for further eligibility assessment. Finally, 4 studies [39,52-54] were retained for the review. The list of excluded publications (n=45) and the reasons for exclusion are provided in Multimedia Appendix 3. We excluded records that are not focused on settings other than LTCFs. We also excluded records that discussed interventions other than ICT-related applications to address SIL.

Studies were conducted in Germany [52], the United States [53], France [39], and the United Kingdom [54]. The following different types of LTCFs were included: nursing homes, LTC homes, and assisted living facilities.

The following designs were used in this study: implementation study [52], observational trial, collaborative action research [54], and cross-sectional study [39,53]. The included studies reached 349 participants ranging from 22 [54] to 132 [39] studies. Several study designs were included in this review and are detailed in Multimedia Appendix 2. We organized the findings of the included studies into themes and further grouped them into natural clusters around certain topics, that is, we identified the following three key promising best practices themes: (1) strategic approach (example), (2) COVID-19 prevention–related interventions (primary and secondary), and (3) COVID-19 free interactive.

### Intervention Type

Numerous types of social-oriented applications were implemented in this review. This includes roughly the use of the ordinary telephone and a variety of platforms for social conversation, that is, in the included studies, the authors dealt with the following platforms: video calls using software, such as Skype, Zoom, and FaceTime [54]; robot temi embedding videotelephony [52]; telephone or video [39]; and desktop computers, laptops, tablets, smartphones, the internet, social media platforms, and videoconferencing [55]**.**

### Focus Area of the Technology

Several types of tools mediating conversations between families and older adults were used in the included studies. The first category of implementation studies in which the intervention object was implemented for 2 to 8 months included Skype [52] and several other types of commercial applications such as FaceTime, Skype, and Zoom [54]. The second batch of studies used a cross-sectional approach to portray the use of ICT-based applications in the context of COVID-19 by older adults to maintain the connection between families and LTCF residents [39,53]. Finally, a third group included the use of robot technology incorporating remote communication systems through video telephony.

### Use of the temi Robot

Follmann et al [52] implemented the use of robots to establish connections between families and residents. These robots have some features that allow the residents to establish connections with relatives through embedded Skype applications. temi served as a companion to the residents. A comparison was made between the 2 study sites. The use of temi resulted in a significant decrease in loneliness compared with the control group, in which residents had no contact (*P*=.01). Several benefits have been demonstrated with the use of temi. For residents, temi’s implementation advantage extends beyond social networking mediation. Because of its autonomy, it rules out the risk of infection with controllable and comfortable operating features (comfort and quality of voice), to cite a few. For relatives, it allows for the continuity of contact with both residents and nursing staff. The absence of financial burden and the risk of infection were also reduced with the implementation of this robot. For the nursing staff, temi offered direct contact with relatives. The possibility of having temi autonomously in a quarantine room, the absence of a need for its supervision, and its ease of disinfection were all factors that facilitated its adoption by the staff.

### The Use of Ordinary Telephone Versus Video Call

The study by Sacco et al [39] compared the use of telephones by older adults in LTCFs versus acute geriatric care. Older adults in an acute geriatric care unit, LTCF, and nursing home found it easier to use a telephone independently compared with video-enabled technology (video calls). Participants reported ease of use of the telephone (73/132, 55.3%) compared with video calls (59/132, 44.7%). Moreover, patients hospitalized in the acute geriatric care unit were more often satisfied with video communication (73/79, 92%) than residents of the LTC and nursing homes (20/27, 74%; *P*=.02), although the latter’s satisfaction experience with video calls was still high.

### Virtual Remote Communication

The study by Schuster and Cotten [53] reported high rates of benefits of socializing virtually with family members (26/34, 77%) and friends (16/34, 47%). However, barriers were identified, such as time to assist residents with the technology, issues related to device maintenance and repair, and residents’ fear of sharing communication devices because of possible contamination (9/34, 26%).

### Effects of Technology on SIL

The study by Zamir et al [54] focused on intergroup video call sessions between residents of 3 care homes and demonstrated that video calls for socialization helped residents to “pass the time,” and gave them “something to do.” The themes generated included feelings of happiness, having a space for expression, and other social activities that allowed older adults to fill their spare time. The following excerpts demonstrate the use of ICT and the expressive feelings of older adults during the COVID-19 pandemic on the topic of their social life. First, the LTCF managers aimed to switch the social activity from in-person (before the COVID-19 pandemic) to a virtual format with an interesting consequence. The following excerpt is an illustration of residents expressing their feelings of happiness: “...With this (quiz), we spoke about our lives and even when I used to live in the country because [resident] also did. I got to share with them... someone new who is happy to hear!” In addition, the following resident from the same study [54] laid out the fact that remote communication in such a situation gave them an opportunity to chat and beyond to share their memories, as portrayed in the following quotation:

When I first came across ... it was amazing ... felt like such an expert! This box was able to communicate from up there ... but now yes it is similar but the technology has changed. Had we been able to see faces then ... well I doubt we could have it was too old.

Another resident added:

Good to see them face-to-face, something to do ... I know it’s not good to speak to people you don’t know ... but ... she’s a talker. Maybe it’s good to use on certain occasions when with friends something to see. I don’t have a house or wife and the years go by now.

### Effects of Technology on Other Collateral Outcomes

The use of the temi humanoid robots in the Follamn et al [52] study showed the importance of this technology for every group, including families, staff members, and older adults. temi’s main purpose is to reduce SIL through chatting. It can be remotely driven; therefore, this technology can reduce some communicable diseases by reducing infectious contact between the staff members and LTCF residents. temi also offers the possibility of being autonomous in a quarantine room, does not require supervision, and is easily disinfected. Finally, temi was seen to be a useful tool for employees in the study by Follmann et al [52].

### Quality Appraisal

To address the variation in study designs, we appraised the quality by using the Specialist Unit for Review Evidence (SURE) critical appraisal checklists [56], as reported by other authors [57]. All 12 items were critically considered to offer an in-depth appraisal of quality. We found it useful to portray the quality of all the components of each study (Multimedia Appendix 4 [39,52-54]). Of these studies, one lasted 78 days [52] and another lasted 8 months [54]. The 4 included studies [39,52-54] used a quantitative and participatory design. All studies implemented nonprobabilistic sampling. Studies unclearly reported participant sampling, and their size was insufficiently described. None of the 4 studies reported any attempt to blind participants from the intervention outcomes being examined or disclose information about whether assessors were aware of the intervention. Only 1 study [52] included a control group; Zamir et al [54] presented a control-like group, and 2 studies used a cross-sectional design [39,53]. The authors did not explain how a certain location, nursing home, or community was selected and why. Finally, although the studies mentioned the number of participants, none of them described how the study size was determined.

## Discussion

### Principal Findings

We found that ICT-related applications were used to ensure connectedness in addressing SIL in LTCFs during the COVID-19 pandemic. There were 3 main categories of ICT: (1) video calls; (2) robot systems; and (3) ordinary telephone, internet, and social media platforms.

To date, SIL is known to be one of the most prevalent issues associated with the aging population trend [58]. This systematic review aimed to portray the available evidence of ICT use in addressing SIL in LTCFs during the COVID-19 pandemic compared with SIL in the long term before the pandemic [59]. Overall, 4 studies were included [39,52-54].

On the basis of the results of this review, we can acknowledge that few studies have been published so far on the topic of ICT use in LTCFs in the context of COVID-19. There is wide heterogeneity in the quality of the studies assessed by the SURE checklist, possibly because of the complex nature of the proposed tool, the design, the duration of the implementation, or the weak sample size.

As stated by Seifert et al [60], the COVID-19 pandemic has incurred a double burden for the residents of LTCFs. First, a large proportion of them were naturally excluded from digital services because of technological illiteracy or their lack of necessary devices and network connectivity [60]. When considering the included studies, there was a clear positive outcome in the few proposed tools in terms of addressing SIL [52,54].

The use of digital tools is a new paradigm for older adults, notably those located in LTCFs, as most of those being relocated to these congregate settings are either older in age or living with disabling comorbidities [61]. Therefore, they tend to have more comorbidities and frailty, affecting their ability to engage with digital tools or learn new associated competencies. Notably, dementia, which affects praxis, language, knowledge acquisition, and mood, is highly prevalent among LTCF residents [62]. Apart from these challenges, our results have shown that some older adults remain averse to digital technology, preferring to use an ordinary telephone [63]. The use of available telephones to reach out to families of older adults, according to the US federal government policy, is mandatory. In several studies [64-66], preference was given to the ordinary telephone to take news of residents. This reluctance might be associated not only with the perception of the complexity of digital tools [67] but also with the resistance to change [68] and difficulties adjusting to change with advancing age and prevalence of cognitive decline. Another reason highlighted in the literature is that, in the context of some group activities with coresidents, some older adults have expressed discomfort and reluctance to open their camera [54].

All these aspects must be considered in the future. First, current older adults face e-technology issues that are overwhelming whereas other older adults are enthusiastic about adopting technology even with limited skills. Growing older tends to be associated with limited digital literacy, but baby boomers are now approaching old age. Worldwide, baby boomers reach retirement age and work longer [69,70] with innovative labor market policies that prolong the working period [71]. Second, as shown in the review, the use of digital technologies is rapidly emerging as an important avenue in LTCFs. Humanoid robot technology is a new innovative avenue for geriatric institutions [72] with multiple purposes, offering live interactions, games, and other friendly companionship features. More research is currently being conducted in Japanese laboratories in the field of robotics to improve human well-being, particularly in old age. They are fraught with a simultaneous risk of 2 major issues: social isolation and nursing care personnel shortage. The avenue of robot technology offers recreational programs to promote communication among older adults in LTCFs [73]. Recreational activities, almost the core activity in an LTCF, subsequently improve the QOL of older adults. Nevertheless, the consequences of robot implementation require upfront capital investment in human resources, equipment, and infrastructure. Overall, the current results on the humanoid robot avenue are beneficial for older adults [73].

In the LTCFs, the added value of the humanoid robot goes beyond serving as a communication tool between older adults and their families. It could also mitigate the risk of communicable disease transmission, as discussed earlier, and can be remotely incorporated. Nevertheless, digital technology is accompanied by limitations. For example, those with dementia tend to have problems with digitally implying tools [15].

This review did not strictly adhere to standard Cochrane methodology. We did not consider gray literature, preprints, conference abstracts, and proceedings. Although the literature published in English and French was included, we recognize the limitations associated with the fact that we ignored publications in other languages (eg, Chinese, Portuguese, and Arab and North Germanic languages). We also planned to exclude hospitalized older adults from the study. However, the study by Sacco et al [39] was retained by the research team because the participants belonged to the same institution (geriatric acute care unit and in the LTC unit and nursing home, University Hospital of Angers, France). Finally, the review included very few studies, despite the tremendous scientific productivity of the COVID-19 pandemic. We hypothesized that many projects are ongoing.

### Recommendations for Policy, Practice, and Future Research

Although heightened during the pandemic, SIL was a pervasive issue in LTCFs in the pre–COVID-19 pandemic era. The findings of this study have important implications for older adults’ QOL agenda for first-line managers in LTCFs and policy makers. Indeed, ICT-related application use offers a convincing perspective for strengthening social connections between families and their loved ones to reduce SIL. The latter is a serious public health concern, such that maintaining LTCF residents’ social capital, namely social sustained connection, is paramount to their QOL. Moreover, ICT platforms (eg, video calls) offer practical means in the post–COVID-19 pandemic era to mitigate the consequences of SIL. Information technology infrastructures (eg, the internet) are acutely lacking—in Canada, for instance [74]—and are therefore needed in 24-hour residential LTC (herein nursing homes in Canada) as defined by Health Canada [75]. Besides crises such as the COVID-19 pandemic, seasonal influenza and gastrointestinal virus outbreaks in nursing homes are very common [76] and often lead to visit bans, although socialization must continue remotely because the goal of LTCFs’ care is to prioritize QOL. This includes QOL at the end of life, avoidance of distress, transfers to the hospital, invasive investigation and interventions, and peaceful death. Because of the lack of available studies, more research is needed to update these review findings and validate the effectiveness of ICT-related applications in combating SIL in LTCFs.

### Conclusions

To the best of our knowledge, this study is certainly one of the first systematic reviews examining the effect of the use of ICT social applications for the purpose of reducing SIL in LTCFs during the COVID-19 pandemic. Undoubtedly, virtual communication has become a new avenue for connecting people, particularly those more at risk of SIL. This is the case for older adults who relocated to congregate settings. It has been the forefront tool used by families to keep in touch with older adults in LTCFs throughout the multiple lengthy and deadly waves of the COVID-19 pandemic. Not reiterating the potential issues of e-technology, some powerful improvements, such as humanoid robots, are coming to the ground. The review has shown positive effects in terms of social connectivity, as well as acceptance by staff members who value its potential to mitigate infectious contact because of its ease of disinfection.

The findings of this systematic review draw attention to the relevant stakeholders of health systems, notably those involved with LTCFs, to address SIL as an urgent and emerging public health issue. This review, the first of the COVID-19 pandemic era, is an initial step to inform policy makers of the need for higher-quality programs for interventions addressing SIL with a special place given to virtually and technologically enabled social interactions. SIL is a socially complex concern in the modern and aging world; therefore, it necessitates a multisectoral approach that includes families, health care workers, managers, and policy makers.

## Appendix

Multimedia Appendix 1 Search strategy.

Multimedia Appendix 2 Characteristics of included studies.

Multimedia Appendix 3 List of excluded studies and reasons.

Multimedia Appendix 4 Critical appraisal of quality—Specialist Unit for Review Evidence (SURE) checklist.

## Abbreviations

FC: family caregiver
ICT: information and communication technology
LTC: long-term care
LTCF: long-term care facility
PI: principal investigator
PICO: Population, Interventions, Comparators and designs, Outcomes
PRISMA: Preferred Reporting Items for Systematic Reviews and Meta-Analyses
QOL: quality of life
SIL: social isolation and loneliness
SURE: Specialist Unit for Review Evidence

## Data Availability

The data sets generated and analyzed during this study will be made publicly available, as requested by the funding institution. All requests should be addressed to the corresponding author, as data will be stored on a secured server of the University of Ottawa.
