# Supplementary material for: Strengthening Social Capital to Address Isolation and Loneliness in Long-Term Care Facilities During the COVID-19 Pandemic: Systematic Review of Research on Information and Communication Technologies
Source: JMIR Aging. 2023 Aug 14;6:e46753. doi: 10.2196/46753 (PMC10463087; doi:10.2196/46753)
Supplement: Multimedia Appendix 1 [file aging_v6i1e46753_app1.docx]

**Annex 1** – Search strategy

**Annex 1.1** Ovid-Medline

| N0 | Query equation |
| --- | --- |
| #1 | (lonel* or 'social connect*' 'social exclusion' or 'social exclusions' or 'social isolation' or  connectedness or 'social distanc*' or aloneness or solitude or 'Seclu* or confin* or separat* or quarantine* or remote* or 'emotional isolation' OR Quarantine).ab,kw,ti. |
| #2 | exp Loneliness/ or exp Quarantine/ or exp Social Isolation/ |
| #3 | (isolat* or deprivation or network or support).ab,kw,ti. and social.ab,kw,ti. |
| #4 | #1 OR #2 OR #3 |
| #5 | ('Assisted-Living Facilit*' or 'Homes for the Aged' or 'Nursing Home*' or Geriatrics or 'Housing for the Elderly' or 'homes for the aged' or 'Housing for the Elderly').ab,kw,ti. |
| #6 | ('Long-Term Care' or LTC or 'long term care' or "longterm care' or long-term-care or Geriatrics or 'Older Adult*' or elde* or senior or aged or retirement or "assisted living').ab,kw,ti. AND (home* or facilit* or residen* or lodg*).ab,kw,ti. |
| #7 | exp residential facilities/ or exp assisted living facilities/ or exp homes for the aged/ or exp skilled nursing facilities/ or exp intermediate care facilities/ or exp Nursing Homes/ OR exp Housing for the Elderly/ or exp Long-Term Care/ |
| #8 | #5 OR #6 OR #7 |
| #9 | (coronavirus or COVID-19 or SRAS-COV2 OR '2019 novel coronavirus' or '2019 novel coronaviruses' or '2019-ncov' or 'covid 19 virus' or 'covid-19 virus' or 'covid-19 viruses' or 'coronavirus disease 2019 virus' or 'sars cov 2 virus' or  'sars coronavirus 2' or 'sars-cov-2' or 'sars-cov-2 virus' or 'sars-cov-2 viruses').ab,kw,ti.= 288011 |
| #10 | ('Digital technology' OR Zoom OR facebook OR 'information technology' OR Skype OR 'FaceTime' or on-line or online or web-based or 'webbased' or 'web based' or 'world wide web' or internet or 'Cellular Phone*' or 'mobile phone*' or smartphone* or smart phone* or cell phone* or iPhone* or video call* or videoconferenc* or video conferenc* or videophone* or 'information communication technolog*' or "ICT*" or 'social media').ab,kw,ti |
| #11 | (virtual or digital).ab,kw,ti and (communit* or network*).ab,kw,ti |
| #12 | exp Cell Phone/ or exp smartphone/ OR exp exp 'Social Support'/ or exp Online Social Networking/ or exp Social Networking/ or exp Social Media/ or exp Internet/ or exp Digital Technology/ or exp Computers/ or exp Information Technology/ or Online Systems/ or exp Online Social Networking/ or exp Computer Communication Networks/ or exp videoconferencing |
| #13 | #11 or #12 or #13 |
| #14 | #4 AND #8 AND #10 AND #13 |

**Annex 1.2** Cochrane Library (Wiley)

| N0 | Query equation |
| --- | --- |
| #1 | (lonel* or 'social connect*' 'social exclusion' or 'social exclusions' or 'social isolation' or  connectedness or 'social distanc*' or aloneness or solitude or 'Seclu* or confin* or separat* or quarantine* or remote* or 'emotional isolation' or Quarantine).ab,kw,ti. |
| #2 | MeSH descriptor: [Loneliness] explode all trees OR MeSH descriptor: [Quarantine] explode all trees OR MeSH descriptor: [Social Isolation] explode all trees |
| #3 | (isolat* or deprivation or network or support).ab,kw,ti. and social.ab,kw,ti. |
| #4 | #1 OR #2 OR #3 |
| #5 | ('Assisted-Living Facilit*' or 'Homes for the Aged' or 'Nursing Home*' or Geriatrics or 'Housing for the Elderly' or 'homes for the aged' or 'Housing for the Elderly').ab,kw,ti. |
| #6 | ('Long-Term Care' or LTC or 'long term care' or 'longterm care' or long-term-care or Geriatric* or 'Older Adult*' or elde* or senior or aged or retirement or 'assisted living').ab,kw,ti. AND (home* or facilit* or residen* or lodg*).ab,kw,ti. |
| #7 | residential facilities or assisted living facilities or homes for the aged or skilled nursing facilities or intermediate care facilities or Housing for the Elderly or nursing home or nursing homes or nursing homes or Housing for the Elderly or long term care or long term cares |
| #8 | #5 OR #6 OR #7 |
| #9 | (coronavirus or COVID-19 or SRAS-COV2 OR '2019 novel coronavirus' or '2019 novel coronaviruses' or 'covid 19 virus' or 'covid-19 virus' or 'covid-19 viruses' or 'coronavirus disease 2019 virus' or 'sars cov 2 virus' or 'sars coronavirus 2' or 'sars-cov-2' or 'sars-cov-2 virus' or 'sars-cov-2 viruses').ab,kw,ti. |
| #10 | (MeSH descriptor: [Epidemics] explode all trees MeSH descriptor: [COVID-19] explode all trees) AND (MeSH descriptor: [Pandemics] explode all trees OR ) |
| #11 | #9 or #10 |
| #12 | ('Digital technology' OR Zoom OR facebook OR 'information technology' OR Skype OR 'FaceTime' or on-line or online or web-based or 'webbased' or 'web based' or 'world wide web' or internet or 'Cellular Phone*' or 'mobile phone*' or smartphone* or smart phone* or cell phone* or iPhone* or video call* or videoconferenc* or video conferenc* or videophone* or 'information communication technolog*' or "ICT*" or 'social media').ab,kw,ti |
| #14 | (virtual or digital).ab,kw,ti and (communit* or network*).ab,kw,ti |
| #15 | Cell Phone or cell phones or cell phones or smartphone or Social Support or Social Supports or Online Social Networking or Social Networking or Social Media or Internet or Digital Technology or Computers or Information Technology or Online Systems or Online Social Networking or Computer Communication Networks or videoconferencing |
| #16 | #12 or #13 or #14 or #15= |
| #17 | #4 AND #8 AND #11 AND #16 |
|  |  |

**Annex 1-3** CINAHL (EBSCO)

| N0 | Query equation |
| --- | --- |
| #1 | TI ( lonel* or "social connect*" "social exclusion" or "social exclusions" or "social isolation" or connectedness or "social distanc*" or aloneness or solitude or Seclu* or confin* or separat* or quarantine* or remote* or 'emotional isolation' or Quarantine ) OR AB (lonel* or "social connect*" "social exclusion" or "social exclusions" or "social isolation" or connectedness or "social distanc*" or aloneness or solitude or Seclu* or confin* or separat* or quarantine* or remote* or 'emotional isolation' or Quarantine) |
| #2 | (MH "Loneliness (Iowa NOC)") OR (MH "Revised UCLA Loneliness Scale") OR (MH "Risk for Loneliness (NANDA)") OR (MH "Loneliness") OR (MH "Quarantine") OR (MH "Social Isolation (NANDA)") OR (MH "Social Isolation (Saba CCC)") OR (MH "Social Isolation+") |
| #3 | [TI ( isolat* or deprivation or network or support ) OR AB ( isolat* or deprivation or network or support ) AND (TI social OR AB social)] |
| #4 | #1 OR #2 OR #3 |
| #5 | TI ( 'Assisted-Living Facilit*' or 'Homes for the Aged' or 'Nursing Home*' or Geriatrics or 'Housing for the Elderly' or 'homes for the aged' or 'Housing for the Elderly' ) OR AU ( 'Assisted-Living Facilit*' or 'Homes for the Aged' or 'Nursing Home*' or Geriatrics or 'Housing for the Elderly' or 'homes for the aged' or 'Housing for the Elderly' ) |
| #6 | [TI ( "Long-Term Care" or LTC or "long term care" or "longterm care" or long-term-care or Geriatric* or "Older Adult*" or elde* or senior or aged or retirement or "assisted living" ) OR AU ( "Long-Term Care" or LTC or "long term care" or "longterm care" or long-term-care or Geriatric* or "Older Adult*" or elde* or senior or aged or retirement or "assisted living" ) AND TI ( home* or facilit* or residen* or lodg* ) OR AU ( home* or facilit* or residen* or lodg* )] |
| #7 | (MH "Residential Facilities+") OR (MH "Housing for the Elderly") OR (MH "Nursing Homes+") OR (MH "Assisted Living") AND (MH "Housing for the Elderly") OR (MH "Long Term Care") |
| #8 | #5 OR #6 OR #7 |
| #9 | (coronavirus or COVID-19 or SRAS-COV2 OR '2019 novel coronavirus' or '2019 novel coronaviruses' or '2019-ncov' or 'covid 19 virus' or 'covid-19 virus' or 'covid-19 viruses' or 'coronavirus disease 2019 virus' or 'sars cov 2 virus' or 'sars coronavirus 2' or 'sars-cov-2' or 'sars-cov-2 virus' or 'sars-cov-2 viruses' ) OR AB ( Pandemi* or epidemi* or andemi* or Outbreak).ab,kw,ti. or (coronavirus or COVID-19 or SRAS-COV2 OR '2019 novel coronavirus' or '2019 novel coronaviruses' or '2019-ncov' or 'covid 19 virus' or 'covid-19 virus' or 'covid-19 viruses' or 'coronavirus disease 2019 virus' or 'sars cov 2 virus' or 'sars coronavirus 2' or 'sars-cov-2' or 'sars-cov-2 virus' or 'sars-cov-2 viruses' ) |
| #10 | [(MH "COVID-19") OR (MH "COVID-19 Pandemic") OR (MH "SARS-CoV-2")] |
| #11 | #9 and #10 |
| #12 | TI ( 'Digital technology' OR Zoom OR facebook OR 'information technology' OR Skype OR 'FaceTime' or on-line or online or web-based or 'webbased' or 'web based' or 'world wide web' or internet or 'Cellular Phone*' or 'mobile phone*' or smartphone* or smart phone* or cell phone* or iPhone* or video call* or videoconferenc* or video conferenc* or videophone* or 'information communication technolog*' or "ICT*" or 'social media' ) OR AB ( 'Digital technology' OR Zoom OR facebook OR 'information technology' OR Skype OR 'FaceTime' or on-line or online or web-based or 'webbased' or 'web based' or 'world wide web' or internet or 'Cellular Phone*' or 'mobile phone*' or smartphone* or smart phone* or cell phone* or iPhone* or video call* or videoconferenc* or video conferenc* or videophone* or 'information communication technolog*' or "ICT*" or 'social media' ) |
| #14 | TI ( (virtual or digital) and (communit* or network*) ) OR AB ( (virtual or digital) and (communit* or network*) ) |
| #15 | (MH "Facebook") OR (MH "Videoconferencing+") OR (MH "Online Social Networking") OR (MH "Social Networks") OR (MH "Social Networking") OR (MH "Health Information Networks") OR (MH "Online Services") OR (MH "Online Social Networking") OR  (MH "Information Technology+") OR (MH "Digital Technology+") OR (MH "Internet+") OR (MH "Social Media+") OR (MH "Online Social Networking") OR (MH "Social Networking+") OR (MH "Support, Psychosocial+") OR (MH "Computer Communication Networks+") OR (MH "Social Support (Iowa NOC)") OR (MH "Smartphone") OR  (MH "Cellular Phone+") |
| #16 | #12 or #13 or #14 or #15 |
| #17 | #4 AND #8 AND #11 AND #16 |

**Annex 1-4** APAPsycINFO(OVID)

| N0 | Query equation |
| --- | --- |
| #1 | (lonel* or "social connect* social exclusion" or "social exclusions" or "social isolation" or connectedness or "social distanc*" or aloneness or solitude or Seclu* or confin* or separat* or quarantine* or remote* or 'emotional isolation' or Quarantine).ab,id,ti. |
| #2 | social isolation/ or exp social deprivation/ or exp patient seclusion/ or exp quarantine/ or exp social anxiety/ or exp social exclusion/ or oneliness/ or exp emotional states/ or exp abandonment/ or exp homesickness/ |
| #3 | [(isolat* or deprivation or network or support).ab,id,ti AND ("isolat*".ab,id,ti.)] |
| #4 | #1 OR #2 OR #3 |
| #5 | ("Assisted-Living Facilit*" or "Homes for the Aged" or "Nursing Home*" or Geriatrics or "Housing for the Elderly" or "homes for the aged" or "Housing for the Elderly").ab,id,ti. |
| #6 | [("Long-Term Care" or LTC or "long term care" or "longterm care" or long-term-care or Geriatric* or "Older Adult*" or elde* or senior or aged or retirement or "assisted living").ab,id,ti. AND (home* or facilit* or residen* or lodg*).ab,id,ti.] |
| #7 | long term care/ or exp health care services/ or exp treatment duration/ or exp home care/ or exp nursing home residents/ or exp nursing homes/ or exp palliative care/ or geriatric psychiatry/ or exp geriatrics/ or exp psychiatry/ or exp aging/ or exp geriatric psychotherapy/ or exp gerontology/ or retirement communities/ or nursing homes/ or residential care institutions/ or retirement communities/ or exp nursing homes/ or exp residential care institutions/ or retirement communities/ or exp nursing homes/ or exp residential care institutions/ or residential care institutions/ or exp assisted living/ |
| #8 | #5 OR #6 OR #7 = |
| #9 | TI ( Pandemi* or epidemi* or andemi* or Outbreak).ab,ti. AND (coronavirus or COVID-19 or SRAS-COV2 or '2019 novel coronavirus' or '2019 novel coronaviruses' or '2019-ncov' or 'covid 19 virus' or 'covid-19 virus' or 'covid-19 viruses' or 'coronavirus disease 2019 virus' or 'sars cov 2 virus' or 'sars coronavirus 2' or 'sars-cov-2' or 'sars-cov-2 virus' or 'sars-cov-2 viruses').ab,id,ti. |
| #10 | exp coronavirus/ |
| #11 | #9 and #10 |
| #12 | ('Digital technology' or Zoom or facebook or 'information technology' or Skype or 'FaceTime' or on-line or online or web-based or 'webbased' or 'web based' or 'world wide web' or internet or 'Cellular Phone*' or 'mobile phone*' or smartphone* or smart phone* or cell phone* or iPhone* or video call* or videoconferenc* or video conferenc* or videophone* or 'information communication technolog*' or "ICT*" or 'social media').ab,id,ti. |
| #14 | (virtual or digital).ab,id,ti. and (communit* or network*).ab,id,ti. |
| #15 | digital technology/ or exp digital information/ or exp digital video/ or exp electronic communication/ or exp mobile technology/ or exp streaming technology/ or exp wireless technologies/ or exp "information and communication technology"/ or exp digital technology/ or exp health information technology/ or  online community/ or exp online social networks/ or internet/ or exp social media/ or exp telecommunications media/ or mobile phones/ or exp mobile devices/ or exp smartphones/ or exp text messaging/ or exp wireless technologies/ or  smartphones/ or mobile phones/ or mobile applications/ or text messaging/ or video-based interventions/ or exp digital video/ or exp videoconferencing/ |
| #16 | #12 or #13 or #14 or #15= |
| #17 | #4 AND #8 AND #11 AND #16 = 792 |

**Annex 1-5** Web of Science

| N0 | Query equation |
| --- | --- |
| #1 | **TS=(lonel* or "social connect* social exclusion" or "social exclusions" or "social isolation" or connectedness or "social distanc*" or aloneness or solitude or Seclu* or confin* or separat* or quarantine* or remote* or 'emotional isolation' or Quarantine)** |
| #2 | [**TS=(isolat* or deprivation or network or support)** AND **TS=(isolation)**] |
| #3 | #1 OR #2 OR #3 |
| #4 | **TS=('Assisted-Living Facilit*' or 'Homes for the Aged' or 'Nursing Home*' or Geriatrics or 'Housing for the Elderly' or 'homes for the aged' or 'Housing for the Elderly')** |
| #5 | **TS=(home* or facilit* or residen* or lodg*) AND TS=("Long-Term Care" or LTC or "long term care" or "longterm care" or long-term-care or Geriatric* or "Older Adult*" or elde* or senior or aged or retirement or "assisted living")** |
| #6 | #5 OR #6 OR #7 |
| #7 | **TS=(coronavirus or COVID-19 or SRAS-COV2 OR '2019 novel coronavirus' or '2019 novel coronaviruses' or '2019-ncov' or 'covid 19 virus' or 'covid-19 virus' or 'covid-19 viruses' or 'coronavirus disease 2019 virus' or 'sars cov 2 virus' or 'sars coronavirus 2' or 'sars-cov-2' or 'sars-cov-2 virus' or 'sars-cov-2 viruses)** |
| #8 | TS=('Digital technology' OR Zoom OR facebook OR 'information technology' OR Skype OR 'FaceTime' or on-line or online or web-based or 'webbased' or 'web based' or 'world wide web' or internet or 'Cellular Phone*' or 'mobile phone*' or smartphone* or smart phone* or cell phone* or iPhone* or video call* or videoconferenc* or video conferenc* or videophone* or 'information communication technolog*' or "ICT*" or 'social media') |
| #9 | #12 or #13 or #14 or #15 |
| #10 | #4 AND #8 AND #11 AND #16 |

**Annex 1-6** IEEE Xplore

| N0 | Query equation |
| --- | --- |
| #1 | lonel* OR  "social isolation" OR  aloneness OR  confin* |
| #2 | ("long-term care" OR geriatric* retirement OR "assisted living") AND home* |
| #3 | **coronavirus or COVID-19 or SRAS-COV2** |
| #4 | "Digital technology" OR online or internet |
| #5 | #1 AND #2 AND #3 AND #4 |

**Annex 1-7** Scopus

| No | Query equation |
| --- | --- |
| #1 | TITLE-ABS-KEY ( ( lonel*  OR  "social connect*"  "social exclusion"  OR  "social exclusions"  OR  "social isolation"  OR  connectedness  OR  "social distanc*"  OR  aloneness  OR  solitude  OR  seclu*  OR  confin*  OR  separat*  OR  quarantine*  OR  remote*  OR  'emotional  AND  isolation'  OR  quarantine )  OR  ( ( isolat*  OR  deprivation  OR  network  OR  support )  AND  ( ti  AND  social ) ) ) |
| #2 | TITLE-ABS-KEY  ( ( ( ( "long-term care" OR ltc OR "long term care" OR "longterm care" OR long-term-care OR geriatric* OR "older adult*" OR elde* OR senior OR aged OR retirement OR "assisted living" ) AND ( home* OR facilit* OR residen* OR lodg* ) ) OR ( ( "assisted-living facilit*" OR "homes for the aged" OR "nursing home*" OR geriatrics OR "housing for the elderly" OR "homes for the aged" OR "housing for the elderly" ) ) ) ) |
| #3 | TITLE-ABS-KEY  **(coronavirus or COVID-19 or SRAS-COV2 OR** "**2019 novel coronavirus**" **or** "**2019 novel coronaviruses**" **or 2019-ncov or** "**covid 19 virus**" **or** "**covid-19 virus**" **or** "**covid-19 viruses**" **or** "**coronavirus disease 2019 virus**" **or** "**sars cov 2 virus**" **or** "**sars coronavirus 2**" **or sars-cov-2' or** "**sars-cov-2 virus**" **or** "**sars-cov-2 viruses**") |
| #4 | TITLE-ABS-KEY  ("Digital technology" OR Zoom OR facebook OR "information technology" OR Skype OR FaceTime or on-line or online or web-based or webbased or "web based" or "world wide web" or internet or "mobile phone*" or smartphone* or "smart phone*" or "cell phone*" or iPhone* or "video call*" or "video conferenc*" or videophone* or "social media") |
| #5 | #1 AND #2 AND #3 AND #4 |

**Annex 1-8** ScienceDirect

| No | Query equation |
| --- | --- |
| #1 | TITLE-ABS-KEY ( lonel*  OR  "social connect*"  "social exclusion"  OR  "social exclusions"  OR  "social isolation"  OR  connectedness  OR  "social distanc*"  OR  aloneness  OR  solitude  OR  seclu*  OR  confin* ) |
| #2 | "long-term care" OR ltc OR "long term care" OR "longterm care" OR long-term-care OR geriatric* OR "older adult*" OR elde* OR senior OR aged OR retirement OR "assisted living" ) AND ( home* OR facilit* OR residen* OR lodg* ) ) |
| #3 | TITLE-ABS-KEY  **(coronavirus or COVID-19 or SRAS-COV2 OR** "**2019 novel coronavirus**" **or** "**2019 novel coronaviruses**" **or 2019-ncov or** "**covid 19 virus**" **or** "**covid-19 virus**" **or** "**covid-19 viruses**" **or** "**coronavirus disease 2019 virus**" **or** "**sars cov 2 virus**" **or** "**sars coronavirus 2**" **or sars-cov-2' or** "**sars-cov-2 virus**" **or** "**sars-cov-2 viruses**") |
| #4 | TITLE-ABS-KEY  ("Digital technology" OR Zoom OR facebook OR "information technology" OR Skype OR FaceTime or online or web-based or "web based" or "world wide web" or internet or "mobile phone*" or smartphone* or "smart phone*" or "cell phone*" or iPhone* or "video call*" or "video conferenc*" or videophone* or "social media") |
| #5 | #1 AND #2 AND #3 AND #4 |

**Annex 1-9** Communications & Mass Media Complete

| N0 | Query equation |
| --- | --- |
| #1 | TITLE-ABS-KEY ( lonel*  OR  "social connect*"  "social exclusion"  OR  "social exclusions"  OR  "social isolation"  OR  connectedness  OR  "social distanc*"  OR  aloneness  OR  solitude  OR  seclu*  OR  confin* ) |
| #2 | "long-term care" OR ltc OR "long term care" OR "longterm care" OR long-term-care OR geriatric* OR "older adult*" OR elde* OR senior OR aged OR retirement OR "assisted living" ) AND ( home* OR facilit* OR residen* OR lodg* ) ) |
| #3 | TITLE-ABS-KEY  **(coronavirus or COVID-19 or SRAS-COV2 OR** "**2019 novel coronavirus**" **or** "**2019 novel coronaviruses**" **or 2019-ncov or** "**covid 19 virus**" **or** "**covid-19 virus**" **or** "**covid-19 viruses**" **or** "**coronavirus disease 2019 virus**" **or** "**sars cov 2 virus**" **or** "**sars coronavirus 2**" **or sars-cov-2' or** "**sars-cov-2 virus**" **or** "**sars-cov-2 viruses**") |
| #4 | TITLE-ABS-KEY  ("Digital technology" OR Zoom OR facebook OR "information technology" OR Skype OR FaceTime or online or web-based or "web based" or "world wide web" or internet or "mobile phone*" or smartphone* or "smart phone*" or "cell phone*" or iPhone* or "video call*" or "video conferenc*" or videophone* or "social media") |
| #5 | #1 AND #2 AND #3 AND #4 |

**Annex 1-10** ACM Digital Library

Social isolation * LTC * Covid-19
