# Supplementary material for: Strengthening Social Capital to Address Isolation and Loneliness in Long-Term Care Facilities During the COVID-19 Pandemic: Systematic Review of Research on Information and Communication Technologies
Source: JMIR Aging. 2023 Aug 14;6:e46753. doi: 10.2196/46753 (PMC10463087; doi:10.2196/46753)
Supplement: Multimedia Appendix 2 [file aging_v6i1e46753_app2.docx]

**Table 1 - Characteristics of included studies**

|  | Year | Country | Type of facility | Study aim | **ICT intervention**  Study design | Recruitment |
| --- | --- | --- | --- | --- | --- | --- |
| Follmann et al., 2021 | 2021 | Germany | 2 nursing homes in rural areas (districts of Heinsberg and Euskirchen) & 1 elderly care University Hospital | To analyze whether the temi is used and if it is sufficiently accepted by the elderly, but also the nursing staff. | Implementation study, quantitative research | Data collection took place in the form of questionnaires, which the participants completed independently if possible. |
| Zamir et al., 2020 | 2020 | UK | Three British care homes (labelled C1, C4, C5) with a specialist care type of dementia and frailty | The study aimed to explore whether inter-care home video calls were an acceptable and feasible intervention to reduce loneliness and social isolation for older people, by: 1) assessing the feasibility and acceptability of using video calls to conduct an inter-care home quiz through Skype on Wheels (SoW) and/or Skype TV with older people living in care homes and  2) determining whether non-familial social contact groups of the same age cohort are useful in increasing socialisation. | Collaborative action research (CAR) design | Recruitment of older people and relevant family was facilitated by staff in the care environment. |
| Sacco et al., 2020 | 2020 | France | All older adults consecutively hospitalized in the geriatric acute care unit and in the long-term care and nursing home | To determine which virtual communication method (i.e., telephone call or video call) was preferred by confined older hospital patients and nursing home residents and the variables influencing this preference | Cross-Sectional Survey Study | **Participants** All older adults consecutively hospitalized in the geriatric acute care unit and in the long-term care and nursing home were considered for inclusion in the study |
| Schuster & Cotten, 2022 | 2022 | USA | long-term care (LTC) facilities: (N=70, 12 nursing homes [NHs], and 58 assisted living facilities [ALFs]) | This study explores ICT access use in LTC facilities and how LTC facilities can be adapted to enhance residents' social connections during the COVID-19 pandemic. | Cross sectional (web-based survey) | From November to December 2020, 193 NH and 496 ALF administrators' email contact information was obtained from the South Carolina Department of Health and Environmental Control (DHEC) website. These LTC administrators were invited to complete through email a Qualtrics web-based survey. After the initial email request for participation, follow-up emails were sent on days 3 and 13.  The survey was based on adaptive questions based on the response and took approximately 20 minutes to complete. During the survey, participants were able to review and change their answers. Overall, 70 LTC administrators (12 NHs and 58 ALFs; 1 participant per site) completed the survey.  Participation was voluntary, and LTC administrators who chose to participate in the study clicked "yes" as a sign of agreement and began responding. LTC administrators who chose to participate in the study clicked "yes" as a sign of agreement and started the survey. |

|  | Sample size | Eligibility criteria | ICT tool | Intervention provider | Intervention components | Implementation process |
| --- | --- | --- | --- | --- | --- | --- |
| Follmann et al., 2021 | n=70 volunteers with three possible interventions (non-contact, virtual encounters by means of a robot, and any other contact) participated in the study. | - Informed consent  - Willingness to be contacted.  - For the virtual encounter group (using the temi robot)  - Participants physically and mentally capable of dealing with technology.  - Depending on the nurses assessments, all other interested persons were assigned to the control group. - Based on nurses' assessments, all other interested individuals were assigned to the control group. | Skype application (Skype Technologies SA, Palo Alto, CA, USA) incorporate in Temi robots, video telephony. | - The residents use the ICT tools themselves (temi is used to make video calls, for one hour, with their relatives)  - A volunteer is assigned to make sure that all questionnaires are completed.  - The type of intervention (virtual encounter using temi, non-contact, or alternative (calls, window visits or video call using a tablet)) was noted on the questionnaires by the nursing staff. | The home care robot temi (Medisana GmbH, Neuss, Germany) was used to establish video telephony via the Skype application. The type of intervention such as virtual encounter using temi, non-contact, or alternative (calls, window visits or video call using a tablet) was also used. | - Three temi robots were used simultaneously in two nursing homes as well as a hospital to perform video call between the patients or residents of the facilities and their relatives for a one-hour window.  - For this purpose, Skype (Skype Technologies SA, Palo Alto, CA, USA) was used, and appointments between relatives and residents were coordinated.  - During the appointments, the residents’ relatives used temi to make video calls, , with their relatives in the respective facilities. - The use of temi technology was analyzed with respect to the number of virtual meetings.  - Each volunteer was asked to fill in a questionnaire containing three questions to measure loneliness. The questionnaire also documented whether video telephony via the robot, an alternative contact option (for example, a phone call), or no contact with relatives had taken place. |
| Zamir et al., 2020 | 22 participants from 3 British care homes (8 care home staff who helped to facilitate the video calls and provided feedback). | Home staff and residents  As for the staffs needs to at least have a College or undergraduate degree level, specialist care type dementia and frailty  As for participants: Dementia or Signs of Cognitive Decline, had to be 65+, no need for previous Experience of Video-Calls | Video calls using software such as Skype, Zoom and FaceTime | Care home staff | Skype on Wheels or Skype TV; Video-call equipment available in care home (iPad, Samsung Galaxy tablet, Skype TV, SoW device), Facebook or emails, FaceTime and now Zoom | As for intervention implementation, a semi-structure interview guide for both residents and care staff was developed by the researcher in the first instance.  **1.** Two months prior to the commencement of the study, an initial first test of this activity (session one) was conducted with C1 and C4  **2.** After this first test session, the participating care home agreed to a session once a month on a date of their choosing to begin with, and then once a fortnight towards the end of the study. Eight sessions were conducted in total as part of this study. Dates and times were agreed between the care homes and confirmed with the researcher through text message or email. A reminder call and/or text message was provided to each care home one week and one day before the session by the researcher. If a care home raised concerns such as the technology not working, the researcher visited the care home to test and help resolve technical issues before the next session was due. Sessions were held in the care home lounge before lunch and lasted for approximately one hour. On average there were six residents participating in a session.  **3.**The session typically began with 15–20 min of ‘meet and greet’ where residents could introduce themselves and make small talk to build friendships. After this, the ‘Skype quiz’ would begin with one care home staff member reading aloud the questions. This responsibility would alternate each session to ensure all care home sites had the equal chance to read their questions.  After each session care staff participating provided short verbal feedback or through text message or telephone call to the researcher. |
| Sacco et al., 2020 | 132 | All older adults consecutively hospitalized in the geriatric acute care unit and in the long-term care and nursing home were considered for inclusion in the study. Patients who refused to participate or who were unable to communicate with their relatives for medical reasons were not included in the study. | Telephone or video | Health professionals | Health professionals accustomed to using communication devices visited all eligible patients at least once per day to offer to help them organize their communications with their relatives. | Health professionals accustomed to using communication devices visited all eligible patients at least once per day to offer to help them organize their communications with their relatives. All patients who expressed interest were offered either a telephone call or a video call, and they were clearly informed that they could receive assistance to establish communication if necessary. All cognitively intact patients who objected to any help were considered to |
| Schuster & Cotten, 2022 | 70 (n=58 in Assisted living facilities and n=12 in Nursing homes) | Older adults residing in LTC facilities (assisted living facilities and Nursing homes) in South Carolina | Desktop computers, laptops, tablets, smartphones, the internet, social media platforms, and videoconferencing | Staff members were the link in facilitating video communication between residents and their family members. Staff members scheduled the videoconferencing visits, helped residents to the area in the home where they could participate in the videoconference call, assisted residents in using the tablets, and cleaned the tablet devices after each use. | Staff members scheduled the video conference visits, helped residents get to the area of the home where they could participate in the video conference call, helped residents use the tablets, and cleaned the tablets after each use. | The state of South Carolina has imposed contact limits on residents of health care facilities and long-term care facilities (called community residential care facilities in South Carolina). Administrators were asked to complete an online survey between November and December 2020 to explore access to and use of ICT in long-term care facilities and to determine whether that access and use has changed as a result of the COVID-19 pandemic. LTC administrators were recruited to participate in the Qualtrics survey via email, which included a secure web link to access the survey. After the initial email requesting participation was sent, follow-up emails were sent after 3 days and 13 days. The Qualtrics survey was 20 pages long with 1-3 questions per page, there were adaptive questions based on the response to other items, and the survey took approximately 20 minutes to complete. During the survey, participants could review and edit their responses using a back button. A total of 70 LTC administrators (12 national care facilities and 58 long-term care facilities; one participant per site) completed the Qualtrics survey. |

|  | Duration / Follow up | Other key info | **Characteristics of participants** /Age **/Sex**  No. of participants | Other details | Outcomes | **Study conclusion**  Limitation |
| --- | --- | --- | --- | --- | --- | --- |
| Follmann et al., 2021 | During a two-month period of the pandemic in 2020. The total observation time was between 1 and 78 days. The participants were interviewed between 1 and 37 times. |  | 19 men, 51 women  83 years old on average (range 59 to 98 years)  The questionnaires of 70 participants were collected for the data evaluation. | - 573 questionnaires were included in the evaluation.  - The frequency of robot use increased steadily over the course of the study, and it was regularly used at all facilities during the no-visit weeks (n = 134 times).  - In the hospital, loneliness decreased significantly in patients for whom the robot was used to make contact (p = 0.01). In nursing homes, no demonstrable effect could be obtained in this way, although the feedback from the subjects about the robot was very positive. although the feedback from the users was consistently positive. | Outcome 1: Temi usage was timed daily during the study period. After an initial training period, the number of virtual encounters rapidly increased in all study sites.  Outcome 2: Loneliness score, as tool a short scientifically established questionnaire on loneliness and social isolation by Hughes et al was used.  **1.** 21% of our participants scored higher than 6.  **2.** Within the longitudinal data, no significant changes of the loneliness scores were found.  **3.** The average loneliness score per volunteer and intervention over time: In the hospital group, the loneliness score was significantly lower among patients who used virtual encounters to meet friends and family virtually compared to among the non-contact group (p = 0.01). For the nursing home in Euskirchen and the nursing home in Heinsberg, virtual encounters did not have any relevant effect regarding the loneliness score compared to the other study groups. | - The sample size is small in terms of limitations, so additional studies are needed. In addition, a longer evaluation period would have allowed the long-term influence of a temi intervention to be studied.  - The existence of long-term effects should also be investigated in further studies. Another disadvantage during the study was the irregularity of the temi contact between the volunteers and their relatives. A fixed schedule would have added value to the results, but this is only partially feasible in practice.  ~~-~~ In conclusion, the study shows that video telephony via a self-driving humanoid-like robot is a practical possibility to maintain the social network of nursing home residents or clinic patients in times of pandemic but also beyond, thus combating social isolation.  - The benefits of using a temi robot extend to all user groups, including the elderly, relatives, and nursing staff of institutions. Temi has been used consistently and successfully in the study from the beginning to counteract this phenomenon.  - Based on the consistent positive feedback, Temi was quickly seen as a member of the group and brought joy to the elderly.  - Temi can significantly reduce the social isolation of hospitalized patients. Compared to nursing home residents, these patients have a shorter treatment time, so there is no formation of a fixed social network in hospital institutions.  - Similarly, temi can facilitate contact between residents and their relatives in nursing homes. |
| Zamir et al., 2020 | - 8 months  - After each video session |  | - n=22  - Residents:65+  facilitators:22-50  - Residents’ =5, f=17 | - Previous experience of video calls: residents: yes=2, no=2, facilitators: yes=3, no=5  - Dementia or signs of cognitive decline: residents: n=7, facilitators: n/d  - Physical disabilities: residents: Hearing impaired =12, Visually impaired= 9, Non-verbal= 3, Frailty = 6, facilitators: male=2, female=6 | - Staff feedback revealed that Skype TV was a preferred method for this activity as SoW was not always able to capture and project the full size of the group from one care home to another.  - Staff felt it was too ‘time consuming’ to continuously wheel around SoW between residents during the activity.  - The software SoW worked well during the ‘meet and greet’ part of the activity as this was more individualised. - Staff also reported that reminders a week in advance would prompt them to check the equipment and report any technical issues rather than leaving it too late. this also enabled care Staff to feel ‘more responsible’ towards the intervention equipment by ensuring it was kept somewhere safe, that it had full power (charging battery of iPad or changing battery in Skype TV remote) and that user logins were easily retrievable.  -After session three as observations and care Staff feedback revealed that residents became more eager to video call in the lead up to the next session as winning became ‘our homes pride’. Similarly, each care home had noticeable ‘top star’ residents who were able to answer questions correctly during the quiz. this in turn helped residents from the other home to remember their names, faces and even their backgrounds.  -Peer interactions across care homes improved vastly from session three to session eight as residents began to remember each other and engage in more meaningful small talk, for example, asking about each other’s families, their fashion and the way their care homes were different or similar. | - The study did not measure for the effects of inter socialisation (within the care home) against intra socialisation (across care homes) which could be a significant contributing factor to consider for future trials in deciding the effectiveness of the video-call intervention.  - Inter-care home connection through video calls to reduce feelings of loneliness in residents seems acceptable and a feasible, low-cost model, especially during times of public crisis such as COVID-19 |
| Sacco et al., 2020 | cross-sectional study | / | n=132  88.2 years (SD: 6.2)  female78 (59.1%) | 1. *Capability of independently establishing communication*: 25 (19.1%)  2. *High degree of satisfaction* (Likert scale score ≥5/6): 93 (87.7%) | *Capability of independently establishing communication*: for patients in geriatric acute care unit:  telephone 22 (36%), video call 2 (5%) [p >.001].  High degree of satisfaction (≥5/6):  Telephone 40 (98%), video call 33 (87%) [p.10].  For residents:  telephone 0 (SD 0), video call 1 (SD 7) [p>.99].  *High degree of satisfaction (≥5/6)*: telephone 6(SD50), choices of video 14(SD 93)[ p.02]. **Outcome 1**: degree of satisfaction 6-point Likert scale  1. Patients in the geriatric acute care unit were more frequently able to independently establish communication (24/105, 22.8%) than residents institutionalized in the long-term care and nursing home (1/27, 3.8%, P.03).  **2.** patients hospitalized in the geriatric acute care unit were more often satisfied with the communication (73/79, 92%) than residents of the long-term care and nursing home (20/27, 74%; P.02). | - The study was monocentric, which limits the representativeness of the study population even if we were able to include a relatively high number of participants.  - The results should be interpreted with caution because some confounding factors such as cognition and mood were not assessed. Larger, and if possible prospective, studies should be conducted on different population groups to better understand the need for video calls and their effects on loneliness, social isolation, and quality of life in older adults.  - Older people confined to health care settings were able to complete telephone calls more independently than video calls, and they tended to use telephone calls more often than video calls. The satisfaction degrees were similar with both modalities and even greater with video calls among long-term care and nursing home residents when they were given assistance to establish communication |
| Schuster & Cotten, 2022 | Cross sectional | After sending the initial email request for participation, follow-up emails were sent at 3 days and 13 days | ICTs have mainly been used for videoconferencing with family members (31/36, 86%), friends (25/36, 69%), and health care providers (26/36, 72%). NHs were 10.23 times more likely to purchase ICTs for residents’ use during the COVID-19 pandemic than ALFs (odds ratio 11.23, 95% CI 1.12-113.02; P.04). Benefits of ICT use included residents feeling connected to their family members, friends, and other residents. Barriers to ICT use included staff not having time to assist residents with using the technology, non-functional technology, and residents who do not want to share technology. | n=70  Elderly residents | **Outcome 1**: Most administrators believed that their facility was at least mostly technologically prepared to meet the social distancing needs of their residents that emerged as a result of the COVID-19 pandemic. (37/70, 57%) ;(tool) Online survey ;(timing) during the study period; (data) (37/70, 57%) of the administrators.  Outcome 2: Most of the facilities provided internet and Wi-Fi, but not all of them allowed residents to access the internet and Wi-Fi even though 94% of the facilities  had Wi-Fi access ; (tool) Web-based survey ; (timing) during the study period ;(data) (69/70, 99%) of the facilities provided internet, (66/70, 94%) had access to Wi-Fi, (59/69, 86% compared to 99% of the facilities who had internet access) did not allowed their residents to access the internet, (57/66, 86% compared to 94% who had Wi-Fi access) did not allowed their resident to use Wi-Fi. A total of 33 of the 70 facilities (47%) had a staff member responsible for providing ICT assistance to residents. Moreover, 59% (38/64) of LTC administrators reported that their residents used laptops, 78% (50/64) of LTC administrators reported that their residents used tablet devices, and 96% (61/64) of LTC administrators reported that their residents used smartphones. Some LTC administrators reported that their residents did not use laptops, tablet devices, or smartphones due to lack of need (18/35, 51%) or prohibitive cost (7/35, 20%).  Outcome 3: Since the COVID-19 lockdown pandemic some of the administrators reported an increase in technology spending at their facility. The majority of the LTC facilities reported purchasing ICTs for their residents using facility funds. ALF administrators reported using personal funds, donations, and small business loans. The top three ICTs purchased by LTC administrators for their residents were tablet devices, smartphones, and laptops. The administrators who purchased ICTs during the COVID-19 pandemic reported that their most common reasons for purchasing were to help residents communicate with their family members and enable telehealth and provide a secure communication channel for their staff. Since the COVID-19 lockdown pandemic some of the administrators reported an increase in technology spending at their facility. The majority of the LTC facilities reported purchasing ICTs for their residents using facility funds.  ALF administrators reported using personal funds, donations, and small business loans. The top three ICTs purchased by LTC administrators for their residents were tablet devices, smartphones, and laptops. The administrators who purchased ICTs during the COVID-19 pandemic reported that their most common reasons for purchasing were to help residents communicate with their family members and enable telehealth and provide a secure communication channel for their staff ;(tool) Web-based survey answering open-ended questions ;  (timing) During study ; (data) Since the COVID-19 lockdown pandemic in the LTC facilities 61% (43/70) of LTC administrators reported an increase in technology spending at their facility. The majority (37/70, 53%) of the LTC facilities reported purchasing ICTs for their residents using facility funds (29/37,78%). Moreover, 45% (5/11) of the nursing home administrators reported using Centers for Medicare and Medicaid Services funding grants to purchase ICTs for their residents (which was provided for COVID-19 communicative technology).  Meanwhile, ALF administrators also reported using personal funds, donations, and small business loans. The top three ICTs purchased by LTC administrators for their residents (non mutually exclusive) were tablet devices (27/37, 73%), smartphones (8/37, 22%), and laptops (8/37, 22%). Out of the 35 of the 37 administrators who purchased ICTs during the COVID-19 pandemic reported that the primary reason for purchasing ICTs were to help residents communicate with their family members and also to enable telehealth and provide a secure communication channel for their staff ; (other outcomes) Administrators reported that, on average, 42% (SD 30.4%) of the residents used the technology provided by facilities and 25% (SD 26.4%) of the residents were not able to use the technology provided by the facility due to health or other impairments. The LTC administrators states that residents have predominantly used the newly purchased ICTs for videoconferencing with family members (31/36, 86%), health care providers (26/36, 72%), and friends (25/36, 69%). Residents have also used the ICTs for entertainment such as playing games (10/36, 28%), shopping (9/36, 25%), and searching for information (8/36, 22%). Though most of the LTC facilities did not have a dedicated person to assist residents with technology use, administrators reported that residents mainly learned to use the ICTs with help from LTC staff members (35/36, 97%). | The sample of the study is predominately for profit LTC facilities. Therefore, the data were collected from older adults residing in LTC facilities (assisted living facilities and Nursing homes) in South Carolina, which limits the generalizability of this study. Almost none of the facilities had a dedicated staff person to assist with technology needs at the time of this.  LTCFs should take into account the technological needs of their residents and provide ongoing support to help them maintain their ICT use, as there is an importance of ongoing technical support for older adults to be able to continue to use ICTs over time. The use of ICTs can be a useful tool to help residents of LTC facilities maintain contact with their social ties, both during and outside of a pandemic. during and outside of a pandemic. Therefore, LTC facilities and residents must have ICT to use, residents must have the skills to use ICT, and support must be available to ensure continued use so that residents can enjoy the benefits of their use. use, and support must be available to ensure continued use so that residents can enjoy the benefits of their use. |
