# Supplementary material for: Strengthening Social Capital to Address Isolation and Loneliness in Long-Term Care Facilities During the COVID-19 Pandemic: Systematic Review of Research on Information and Communication Technologies
Source: JMIR Aging. 2023 Aug 14;6:e46753. doi: 10.2196/46753 (PMC10463087; doi:10.2196/46753)
Supplement: Multimedia Appendix 3 [file aging_v6i1e46753_app3.docx]

**Multimedia Appendix 3: List of the 45 studies finally excluded and reasons**

| **NO** | **Title** | **Reason of exclusion** |
| --- | --- | --- |
| 1 | Zhao, X., Fan, J., Basnyat, I., & Hu, B. (2020). Online Health Information Seeking Using "#COVID-19 Patient Seeking Help" on Weibo in Wuhan, China: Descriptive Study. | Not related to SIL and/ or ICT |
| 2 | Zhang, F., & Cheng, S. T. (2022). Benefit-finding buffers the effects of home confinement and centralized quarantine (HCCQ) on late midlife and older adults' mental health during the COVID-19 pandemic. | Not related to SIL and/ or ICT  Wrong setting |
| 3 | Zeghari, R., Guerchouche, R., Tran Duc, M., Bremond, F., Lemoine, M. P., Bultingaire, V., Langel, K., De Groote, Z., Kuhn, F., Martin, E., Robert, P., & König, A. (2021). Pilot Study to Assess the Feasibility of a Mobile Unit for Remote Cognitive Screening of Isolated Elderly in Rural Areas. | Not related to SIL and/ or ICT |
| 4 | Zhiying Yue, David S. Lee, Jun Xiao & Renwen Zhang (2021) Social media use, psychological well-being and physical health during lockdown | Not related to SIL and/ or ICT |
| 5 | Yu, Wild, K., Potempa, K., Hampstead, B. M., Lichtenberg, P. A., Struble, L. M., Pruitt, P., Alfaro, E. L., Lindsley, J., MacDonald, M., Kaye, J. A., Silbert, L. C., & Dodge, H. H. (2021). The Internet-Based Conversational Engagement Clinical Trial (I-CONECT) in Socially Isolated Adults 75+ Years Old: Randomized Controlled Trial Protocol and COVID-19 Related Study Modifications. | Wrong setting |
| 6 | Yang, Rigsby, M., Zhu, X., Lee, C., & Ory, M. (2022). COVID-19 in Long-Term Care Facilities: A Rapid Review of Infection Correlates and Impacts on Mental Health and Behaviors. | Not related to SIL and/ or ICT |
| 7 | Yamamoto, Uchiumi, C., Suzuki, N., Yoshimoto, J., & Murillo-Rodriguez, E. (2020). The Psychological Impact of “Mild Lockdown” in Japan during the COVID-19 Pandemic: A Nationwide Survey under a Declared State of Emergency. | Not related to SIL and/ or ICT |
| 8 | Wiwatkunupakarn, Pateekhum, C., Aramrat, C., Jirapornchaoren, W., Pinyopornpanish, K., & Angkurawaranon, C. (2022). Social networking site usage: A systematic review of its relationship with social isolation, loneliness, and depression among older adults | Not related to SIL and/ or ICT |
| 9 | Wang, Yan, H., Yan, J., & Qian, L. (2021). The Role of Computer Remote Monitoring Technology for Nursing Care in Elderly Breast Cancer Complications. | Not related to SIL and/ or ICT |
| 10 | Wammes, Kolk, Ms., van den Besselaar, M., MacNeil-Vroomen, P., Buurman-van Es, R., & van Rijn, P. (2020). Evaluating Perspectives of Relatives of Nursing Home Residents on the Nursing Home Visiting Restrictions During the COVID-19 Crisis: A Dutch Cross-Sectional Survey Study. | Wrong population |
| 11 | Vincenzo, Hergott, C., Schrodt, L., Rohrer, B., Brach, J., Tripken, J., Shirley, K. D., Sidelinker, J. C., & Shubert, T. E. (2021). Capitalizing on Virtual Delivery of Community Programs to Support Health and Well-Being of Older Adults. | Not related to SIL and/ or ICT |
| 12 | Veiga-Seijo, Miranda-Duro, M. del C., & Veiga-Seijo, S. (2022). Strategies and actions to enable meaningful family connections in nursing homes during the COVID-19: A Scoping Review. | Wrong publication type (REVIEW) |
| 13 | van Dyck, Wilkins, K., Mecca, M., Lane, C., & Conroy, M. (2021). Social Connections for Seniors During COVID-19: An Online Psychoeducation and Peer Support Program. | Wrong publication type (POSTER) |
| 14 | Van Dyck 2020 Combating Heightened Social Isolation of Nursing Home Elders: The Telephone Outreach in the COVID-19 Outbreak Program | Wrong publication type (Brief) |
| 15 | Van der Roest, Prins, M., van der Velden, C., Steinmetz, S., Stolte, E., van Tilburg, T. G., & de Vries, D. H. (2020). The Impact of COVID-19 Measures on Well-Being of Older Long-Term Care Facility Residents in the Netherlands. | Not related to SIL and/ or ICT |
| 16 | Tso, & Park, S. (2020). Alarming levels of psychiatric symptoms and the role of loneliness during the COVID-19 epidemic: A case study of Hong Kong. | Not related to SIL and/ or ICT |
| 17 | Tsai, Cheng, C.-Y., & Shieh, W.-Y. (2020). Effectiveness of laptop-based versus smartphone-based videoconferencing interaction on loneliness, depression and social support in nursing home residents: A secondary data analysis. | Wrong date |
| 18 | Torres, Braga, L. de S., Moreira, B. de S., Sabino Castro, C. M., Vaz, C. T., Andrade, A. C. de S., Bof Andrade, F., Lima-Costa, M. F., & Caiaffa, W. T. (2022). Loneliness and social disconnectedness in the time of pandemic period among Brazilians: evidence from the ELSI COVID-19 initiative. | Wrong setting |
| 19 | S. Thunberg and T. Ziemke (2021). Pandemic Effects on Social Companion Robot Use in Care Homes. | Not related to SIL and/ or ICT |
| 20 | Tan, Liu, G., & Seetharaman, S. K. (2021). Pivoting volunteer engagement activities for older adults in long‐term care facilities and day care centres online during the COVID‐19 pandemic. | Not related to SIL and/ or ICT |
| 21 | Sen, Prybutok, G., & Prybutok, V. (2022). The use of digital technology for social wellbeing reduces social isolation in older adults: A systematic review. | Not related to SIL and/ or ICT |
| 22 | Seifert, Cotten, S. R., & Xie, B. (2021). A Double Burden of Exclusion? Digital and Social Exclusion of Older Adults in Times of COVID-19. | Wrong outcome |
| 23 | Rorai, & Perry, T. E. (2020). An Innovative Telephone Outreach Program to Seniors in Detroit, a City Facing Dire Consequences of COVID-19. | Wrong setting |
| 24 | Romanopoulou, E. D., Zilidou, V. I., Gilou, S., Dratsiou, I., Varella, A., Petronikolou, V., Katsouli, A. M., Karagianni, M., & Bamidis, P. D. (2021). Technology Enhanced Health and Social Care for Vulnerable People During the COVID-19 Outbreak. | Wrong outcome |
| 25 | Rolandi, E., Vaccaro, R., Abbondanza, S., Casanova, G., Pettinato, L., Colombo, M., & Guaita, A. (2020). Loneliness and Social Engagement in Older Adults Based in Lombardy during the COVID-19 Lockdown: The Long-Term Effects of a Course on Social Networking Sites Use. | Not related to SIL and/ or ICT |
| 26 | Rodrigues, Han, C. Q. Y., Su, Y., Klainin‐Yobas, P., & Wu, X. V. (2022). Psychological impacts and online interventions of social isolation amongst older adults during COVID‐19 pandemic: A scoping review. | Wrong outcome |
| 27 | Robič, M., & Rotar Pavlič, D. (2021). COVID-19 and Care for the Elderly in Long-Term Care Facilities: The Role of Information Communication Technology. | Wrong outcome |
| 28 | Raina, Gupta, A., Gupta, U., Singh, U., & Jain, D. (2022). Eldercare helpline: connecting with older people to mitigate the effects of the Covid-19 crisis in Jaipur, India. | Not related to SIL and/ or ICT |
| 29 | Prophater, Fazio, S., Nguyen, L. T., Hueluer, G., Peterson, L. J., Sherwin, K., Shatzer, J., Branham, M., Kavalec, A., O’Hern, K., Stoglin, K., Tate, R., & Hyer, K. (2021). Alzheimer’s Association Project VITAL: A Florida Statewide Initiative Using Technology to Impact Social Isolation and Well-Being. | Not related to SIL and/ or ICT |
| 30 | Peyrusqué, Granet, J., Pageaux, B., Buckinx, F., & Aubertin-Leheudre, M. (2021). Assessing Physical Performance in Older Adults during Isolation or Lockdown Periods: Web-Based Video Conferencing as a Solution. | Not related to SIL and/ or ICT |
| 31 | O’Shea, Finlay, J. M., Kler, J., Joseph, C. A., & Kobayashi, L. C. (2021). Loneliness Among US Adults Aged ≥55 Early in the COVID-19 Pandemic: Findings From the COVID-19 Coping Study. | Not related to SIL and/ or ICT |
| 32 | O’Caoimh, O’Donovan, M. R., Monahan, M. P., Dalton O’Connor, C., Buckley, C., Kilty, C., Fitzgerald, S., Hartigan, I., & Cornally, N. (2020). Psychosocial Impact of COVID-19 Nursing Home Restrictions on Visitors of Residents With Cognitive Impairment: A Cross-Sectional Study as Part of the Engaging Remotely in Care (ERiC) Project. | Not related to SIL and/ or ICT |
| 33 | Noone, Noone, C., McSharry, J., Smalle, M., Burns, A., Dwan, K., Devane, D., & Morrissey, E. C. (2020). Video calls for reducing social isolation and loneliness in older people: a rapid review. | Wrong date |
| 34 | COVID-19 and Social Isolation and Loneliness Trial (2022). https://www.cochranelibrary.com/central/doi/10.1002/central/CN-02362810/full | Wrong setting |
| 35 | Naudé, Rigaud, A.-S., & Pino, M. (2021). Video Calls for Older Adults: A Narrative Review of Experiments Involving Older Adults in Elderly Care Institutions. | Wrong outcome |
| 36 | Lawless, M. T., Archibald, M., Pinero de Plaza, M. A., Drioli-Phillips, P., & Kitson, A. (2020). Peer-to-Peer Health Communication in Older Adults' Online Communities: Protocol for a Qualitative Netnographic Study and Co-Design Approach. | Wrong outcome |
| 37 | Latikka, R., Rubio-Hernández, R., Lohan, E. S., Rantala, J., Nieto Fernández, F., Laitinen, A., & Oksanen, A. (2021). Older Adults' Loneliness, Social Isolation, and Physical Information and Communication Technology in the Era of Ambient Assisted Living: A Systematic Literature Review. | Wrong setting |
| 38 | Eghtesadi M. (2020). Breaking Social Isolation Amidst COVID-19: A Viewpoint on Improving Access to Technology in Long-Term Care Facilities. | Wrong outcome |
| 39 | Chu, C. H., Ronquillo, C., Khan, S., Hung, L., & Boscart, V. (2021). Technology Recommendations to Support Person-Centered Care in Long-Term Care Homes during the COVID-19 Pandemic and Beyond. | Wrong outcome |
| 40 | Choi, H. K., & Lee, S. H. (2021). Trends and Effectiveness of ICT Interventions for the Elderly to Reduce Loneliness: A Systematic Review. | Wrong population |
| 41 | Chen, A. T., Ge, S., Cho, S., Teng, A. K., Chu, F., Demiris, G., & Zaslavsky, O. (2021). Reactions to COVID-19, information and technology use, and social connectedness among older adults with pre-frailty and frailty. | Not related to SIL and/ or ICT |
| 42 | Beogo, I., Sia, D., Tchouaket Nguemeleu, E., Zhao, J., Gagnon, M. P., & Etowa, J. (2022). Strengthening Social Capital to Address Isolation and Loneliness in Long-term Care Facilities During the COVID-19 Pandemic: Protocol for a Systematic Review of Research on Information and Communication Technologies. | Wrong outcome |
| 43 | Beogo, Ramdé, J., Nguemeleu Tchouaket, E., Sia, D., Bationo, N. J.-C., Collin, S., Anne, A., & Gagnon, M.-P. (2021). Co-Development of a Web-Based Hub (eSocial-hub) to Combat Social Isolation and Loneliness in Francophone and Anglophone Older People in the Linguistic Minority Context (Quebec, Manitoba, and New Brunswick): Protocol for a Mixed Methods Interventional Study. | Wrong outcome |
| 44 | Anthony Jnr B. (2021). Implications of telehealth and digital care solutions during COVID-19 pandemic: a qualitative literature review. | Wrong outcome |
| 45 | Loveys, K., Sagar, M., Pickering, I., & Broadbent, E. (2021). A Digital Human for Delivering a Remote Loneliness and Stress Intervention to At-Risk Younger and Older Adults During the COVID-19 Pandemic: Randomized Pilot Trial. JMIR Ment Health, 8(11), e31586. doi: 10.2196/31586 | Wrong setting |
