# Supplementary material for: Strengthening Social Capital to Address Isolation and Loneliness in Long-Term Care Facilities During the COVID-19 Pandemic: Systematic Review of Research on Information and Communication Technologies
Source: JMIR Aging. 2023 Aug 14;6:e46753. doi: 10.2196/46753 (PMC10463087; doi:10.2196/46753)
Supplement: Multimedia Appendix 4 [file aging_v6i1e46753_app4.docx]

**Multimedia Appendix 4: Critical appraisal of quality (SURE checklist)**

|  | Schuster & Cotten, 2022 | Zamir et al, 2020 | Follmann et al, 2021 | Sacco et al. 2020 |
| --- | --- | --- | --- | --- |
| 1. Is the study design clearly stated? | Yes | Yes | Yes | Yes |
| 2. Does the study address a clearly focused question? Consider: Population; Exposure (defined and accurately measured?); Outcomes. | Yes | Yes | Yes | Yes |
| 3. Are the setting, locations and relevant dates provided? Consider: recruitment period; exposure; data collection. | Yes | Yes (study of implementation period not stated) | Yes (study of implementation period not stated) | Yes |
| 4. Were participants fairly selected? Consider: eligibility criteria; sources & selection of participants. | Yes | Unclear | Unclear | Yes |
| 5. Are participant characteristics provided? Consider if: sufficient details; a table is included. | Yes | Yes | Yes | Yes |
| 6. Are the measures of exposures & outcomes appropriate?  Consider if the methods of assessment are valid & reliable. | N/A | Yes | Yes | Yes |
| 7. Is there a description of how the study size was arrived at? | Can't tell | Can't tell | No | Yes |
| 8. Are the statistical methods well described? Consider: How missing data was handled; were potential sources of bias (confounding factors) considered/controlled for. | Yes (how missing data were handled is not stated) | Yes (more qualitative data presented) | Yes (descriptive) | Yes |
| 9. Is information provided on participant eligibility? Consider if following provided: number potentially eligible, confirmed eligible, entered into study | Yes | Yes | Can't tell | Yes |
| 10. Are the results well described? Consider if: effect sizes, confidence intervals/standard deviations provided; the conclusions are the same in the abstract and the full text. | Yes | Yes | Yes | Yes |
| 11. Is any sponsorship/conflict of interest reported? | No | No | Yes (reported) | No |
| 12. Finally...Did the authors identify any limitations and, if so, are they captured above? | Yes (finding generalisation limited) | No (not stated) | Yes (weak sample size, shorter evaluation time) | Yes (monocentric study) |
